# Germination Study of Some Protein-Based Gels Obtained from By-Products from the Leather Industry on Tomato and Pepper Seeds

**DOI:** 10.3390/gels10010075

**Published:** 2024-01-19

**Authors:** Stelica Cristea, Mihaela-Doina Niculescu, Alina Perisoara, Elena Ivan, Maria Stanca, Cosmin-Andrei Alexe, Bianca-Maria Tihauan, Laura Olariu

**Affiliations:** 1 Plant Pathology Departament, University of Agronomical Sciences and Veterinary Medicine, 011464 Bucharest, Romania; stelicacristea@yahoo.com (S.C.); elena.ivan@qlab.usamv.ro (E.I.); 2 Leather Research Department, Research and Development National Institute for Textiles and Leather-Division Leather and Footwear Research Institute, 93, Ion Minulescu Str., 031215 Bucharest, Romania; mihaelaniculescu59@yahoo.com (M.-D.N.); maria.alexandu@gmail.com (M.S.); cosminandrei.alexe@yahoo.com (C.-A.A.); 3 Research Institute, University of Bucharest-ICUB, Splaiul Independenţei, No. 95, District 5, 050095 Bucharest, Romania; ciubuca.b@gmail.com; 4 Academy of Romanian Scientists, 54 Splaiul Independentei, 050094 Bucharest, Romania; ilolariu@yahoo.com

**Keywords:** protein-based gels, biostimulants, amino acids, ecological agriculture

## Abstract

This study aimed to evaluate the biostimulant effects of three protein-based gels, GHC 1-B (20% gelatin (GPU-B) obtained by thermal hydrolysis from residual untanned leather and 80% collagen hydrolysates (HCE-B) obtained by alkaline–enzymatic hydrolysis from residual bovine-tanned leather), GHC 2-B (40% keratin hydrolysate (HKU-B) obtained by alkaline–enzymatic hydrolysis from sheep wool + 40% HCE-B + 20% GPU-B), and GHC 3-B (20% GPU-B + 80% hydrolyzed collagen (HPU-B) obtained by thermal and enzymatic hydrolysis from residual untanned leather). A germination study was carried out on pepper and tomato seeds at concentrations of 1%, 3%, and 10%. As a result of the study, it was found that all three protein-based gels showed a stimulatory effect on the tomato seeds at a 1% concentration, where the Gi (germination index) was ˂100%. The GHC 2-B variant had the highest stimulatory effect (Gi-190.23%). Pepper seeds have proven to be more sensitive to the gel’s composition. The concentration at which it proved to be non-inhibitory (Gi–88.29%) was 1% in the case of GHC 2-B. It was found that the presence of hydrolyzed keratin in the composition can be a plus compared to the other two protein gels tested due to its composition, which is richer in phytonutrient compounds (e.g., sulfur molecules).

## 1. Introduction

The use of biological waste and secondary sources of nutrients to obtain products with biostimulatory properties for plants is of increasing interest to researchers to support ecological agriculture. Currently, there is a need for the development and implementation of measures that are environmentally friendly but also respond to the population’s need to be fed as healthily as possible, which remains one of the most important objectives for agriculture [1]. Thus, using biostimulants based on hydrolyzed proteins from both vegetable and animal sources is one of the most promising measures to achieve these objectives in the agricultural industry [2]. Biostimulators, in general, are substances that intervene with the plant’s metabolism and can be made up of either a single compound or a mixture of substances. Biostimulators can be based on substances of animal or plant origin [3] and can be applied to the soil or foliar. They are presented in liquid, powder, or gel forms. According to Golka et al. [4], the use of biostimulators in the form of a gel helps to maintain the moisture of the plant by extensive water absorption, being mixed with the soil, increasing the water capacity, counteracting water tensions by ensuring humidity for the plants, and reducing the evaporation of the water from the soil [5]. Another study [6] highlights the impact of the application of protein gels on tomato seedlings compared to samples of seedlings not treated with protein gels. After analyzing the leaves of tomato seedlings, it was found that the seedlings treated with the protein gels had a higher content of pigments, carotenoids, total phenolic compounds, and soluble sugars.

Biostimulants based on hydrolyzed proteins present a series of advantages, such as improved plant growth and development, nutritional absorption, and tolerance to abiotic and biotic stresses, and are used in reduced quantities compared to classic chemical fertilizers [7]. Specialized literature contains a series of products and experimental results based on mixtures containing a series of substances with phytostimulatory potential, such as protein hydrolysates, nitrogen, phosphates, potassium compounds, and polyphosphates, which are used in agricultural industries both in field crops and greenhouses. Added value is brought to micro-elements, such as magnesium, copper, zinc, iron, calcium, and manganese when they are associated with protein hydrolysates because they are absorbed faster by plants [8]. Hydrolyzed proteins, regardless of their source, can be obtained from organic waste, which places them in the current development trend of sustainable and ecological agriculture [9]. Protein hydrolysates of animal origin are generally obtained from chicken feathers and conjunctive and epithelial tissues from animals [10], such as sheep, cattle, and pigs, as well as from bone meal, and those of vegetable origin are obtained from a series of vegetable by-products such as alfalfa rewalls, wheat condensate, algae proteins, and carob sprouts [11]. In addition to protein hydrolysates as potential biostimulant products, the following bioactives can also be mentioned: peptides, enzymes, vitamins, substances with hormone effects—seaweed extracts, fulvic acids, and humic acids—and beneficial microorganisms (bacteria fixing nitrogen—Rhizobium, Azotobacter, etc.) [12]. The vast majority of biostimulants based on protein hydrolysates in the market contain products obtained through chemical hydrolysis, and this category includes protein hydrolysates obtained from animal sources. However, there is another less-used category of biostimulants based on protein hydrolysates that are obtained from vegetable sources through enzymatic hydrolysis. Protein hydrolysates from animal sources are generally obtained via chemical or alkaline hydrolysis [13]. The acid hydrolysis process also has several negative aspects as it is carried out at fairly high temperatures, which destroys the peptide bonds, ultimately resulting in a high concentration of free amino acids and the destruction of some beneficial chemical compounds for plants, most of which are amino acids. This can also lead to racemization and/or the transition of free amino acids from the L-form to the D-form, resulting in less potent protein hydrolysates or even toxic effects on plants [14], such as increased salinity. Instead, enzymatic hydrolysis is carried out by proteolytic enzymes without the need for heat treatment. Following this process, peptides of variable lengths and a fairly balanced concentration of amino acids were obtained. They usually target specific peptide bonds, resulting in a combination of amino acids and peptides of variable length, low salinity, and consistent composition [15].

To evaluate the behavior of plants in certain growth environments, several complex analytical techniques can be applied for the identification and quantification of phytostimulants/phytotoxic substances, or seed germination bioassays can be used [16]. The most accurate way of interpreting the effect of an extract or a compound on germinated plants is through the roots’ development because they are responsible for the accumulation and absorption of beneficial or non-beneficial substances [17]. In general, chemical compounds such as amino acids, phenolic compounds, alkaloids, flavonoids, amino acids, and carbohydrates [18] that enter the composition of some extracts at too high concentrations can show a phytotoxic character for plants, which puts them in the category of allelopathic compounds [19]. However, some studies reveal the fact that these compounds can have a stimulating effect on plants at low concentrations [20]. Considering that there is a growing interest in the use of biologically active substrates in the agricultural industry, the objective of this work was to investigate the stimulatory potential of four types of protein hydrolysates extracted from tanned leather of bovine and ovine origin obtained by using acid–thermal, alkaline, and alkaline–enzymatic hydrolysis. The protein hydrolysates obtained from the hydrolysis processes were combined in different proportions, finally obtaining three protein-based gels, GHC 1-B, GHC 2-B, and GHC 3-B. The study of the stimulatory effect was carried out on two types of seeds, tomatoes, and peppers, by applying a germination biotest. In addition, physicochemical characterization studies of protein hydrolysates and amino acid content were carried out using the HPLC chromatographic method, the surface properties of the protein combinations were determined, and the size of the nanometric particles and the distribution in the combination of proteins were measured using dynamic light scattering. The hydrolysates extracted from the leather tanned with chromium were studied a long time ago, and it was found that under extraction conditions similar to those described in this research, the total chromium content ranged between 18 and 54 ppb by atomic absorption spectroscopy [21,22]. Moreover, the previous application studied cereal crops, technical plants, and horticulture and did not signal deficiencies induced by chromium accumulation in plants, on seed germination, on plant roots, on plant seedlings, on above-ground parts of plants, or reduced growth, and the yield of plants [23,24].

## 2. Results and Discussion

### 2.1. Protein Extracts and Their Combination Characterizations

#### 2.1.1. Physical–Chemical Characteristics of the Protein Extracts and Their Combinations

Table 1 shows the results of the physicochemical analyses of the protein extracts and the protein-based gel combinations, which were performed by applying the available standards and the institute’s methods.

The differences between the amino nitrogen content of the collagen hydrolysate made from untanned leather (HPU-B) compared to the hydrolysate obtained from tanned leather (HCE-B) indicate that by destroying the bonds between the tanant and the collagen matrix during hydrolysis, fragments are released as peptides with small sizes, leading to a wider spectrum of small sizes. In previous research [25], it was demonstrated that collagen hydrolysate with an amino nitrogen content greater than 0.55% contains free amino acids (histidine, alanine, glutamic acid, arginine, glycine, leucine, isoleucine, methionine, aspartic acid, valine, and proline) and oligopeptides (phenylalanine/leucine and isoleucine/lysine), important for vegetative biostimulation and plant nutrition.

#### 2.1.2. The Texture Properties of Gelatin

Analytical data according to the Bloom test indicate that GBPU-B gelatin has a medium strength expressed by a maximum force of 200 g. The consistency was evaluated by the CRT (contraction–relaxation–tension) test, defined by a maximum force of 1270.7 g, with the elasticity being inversely proportional to the relaxation of 44.2%, and the adhesion force defined by a minimum force of −5.3 g.

Being a medium-level gelatin, there is no risk of coagulation at lower temperatures or at low dilutions when preparing protein additive mixtures, but it ensures a sufficient content of large polypeptides to provide adhesion for surface applications and a delayed release of protein components with low molecular masses.

#### 2.1.3. The Amino Acid Compositions in Protein Extracts

The percentage content of amino acids in protein extracts is presented in Table 2.

Analyzing the amino acid profile by comparing it with other results obtained in previous research [26,27,28,29], we find that no significant differences were recorded. For gelatin, there are differences of a max. of 1.5% for glycine and arginine concentrations. For collagen hydrolysates, the differences are below 1.0% for all amino acids, and for keratin hydrolysates, the differences are below 2.5% for threonine, glutamine, tyrosine, and arginine content. These slight differences appear due to the degree of previous processing of the leather used for extraction as well as the different extraction conditions. Obtaining protein extracts from by-products of leather processing (downgraded products due to non-compliant characteristics or classified as manufacturing waste) generated some anomalies in the amino acid content, such as the small amounts of cysteine or tryptophan degradation products in the composition of gelatins or in collagen hydrolysate, which come from the traces of hair in the dermis after the liming process.

#### 2.1.4. The Surface Properties of Protein Combinations

Because hydrolyzed protein-based gel combinations will interact with the surfaces of seeds and leaves of horticultural plants, surface properties are important. Surface tension is an important parameter in the foliar application of products for the treatment of seeds or growing plants as it defines the ability of these products to wet the surface on which they are applied, which is an essential condition for the transfer of active substances. Likewise, the contact angles of liquids with the treated surface are important, being a measure of how well a surface is wetted. Since all three gel combinations are aqueous, water was used as a control in the determination of surface tensions and contact angles. A standard glass surface was used to determine the contact angles. The measured surface tensions and contact angles for the protein gel combinations are shown in Table 3 and Table 4, respectively.

As expected, the three compositions have lower surface tension than water due to the combination of water and other substances.

Both the surface tensions of the protein gel compositions, which define hydrophilic mixtures, as well as the static contact angles with values below 90 are favorable for good wetting of a standard surface (glass), which is a first condition for a good substance transfer between the applied protein gel composition and seeds or the plant due to the phenomenon of capillarity.

#### 2.1.5. The Nanometer Particle Size and Distribution in Protein-Based Gels

**Figure 1:**
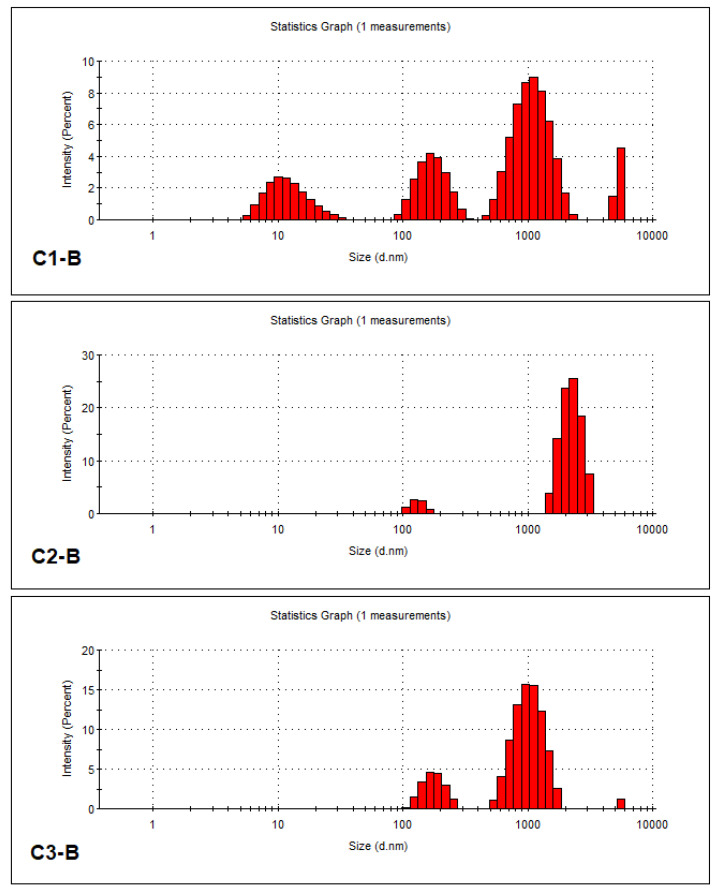
The size of nanometric particles from the protein-based gels: C1-B for GHC1-B; C2-B for GHC2-B; C3-B for GHC3-B.

In Figure 1, the sizes of nanometer particles from all three protein-based gels described in Part 4 are shown as histograms.

The percentage size distribution of nanometric particle populations in the protein-based gel variants is presented in Table 5.

It is noted that the reflected light intensity measurements indicate small particle populations in the 0–10 nm and 10–100 nm ranges for the GHC 1-B gel variant with collagen hydrolysates obtained from tanned bovine leather, medium particle populations located in the 100–1000 nm range, which are dominant for the GHC 1-B and GHC 3-B gel variants that contain collagen hydrolysates, and large particles in the 1000–6000 nm range, which are predominant in the GHC 2-B protein gel variant that contains keratin hydrolysate. Also, for all analyzed protein gel variants, the stability is evaluated as good, with Zeta potential values of −4.64 mV for GHC 1-B, −10.1 mV for GHC 2-B, and −4.57 mV for GHC 3-B. These data suggest that GHC 1-B protein gel could be more suitable for the treatment of seeds, to stimulate germination, and to have a composition of free amino acids and oligopeptides; GHC 2-B and GHC 3-B protein gel variants would be more suitable for the treatment of horticultural plants in vegetation, having a potential retardation release of oligopeptides and amino acids from large polypeptide particles.

### 2.2. Seed Germination Bioassay

#### 2.2.1. Results Regarding the Percentage of Germination (GP%)

The phytotoxic and/or phytostimulant potential is determined by interpreting the value of the germination index—Gi—and this, in turn, is obtained by interpreting the behavior of the seeds during the germination study, which had the following parameters: the relative germination of the seeds (RSG) and the relative growth of roots (RRG) [30]. According to Bernal and his collaborators [29], a new product with phytostimulant potential for plants should be tested and verified before being applied in the field and greenhouses because too high a concentration of active substances could have an inhibition effect on seed germination, plant growth, and development, on the soil environment due to the decrease in nitrogen and oxygen supply, and the presence of possible phytotoxic compounds. The GP% obtained, following the treatment of pepper and tomato seeds with the three types of protein-based gels marked GHC 1-B, GHC 2-B, and GHC 3-B at concentrations of 1%, 3%, and 10% on the seed’s pepper, fell between 100–16.66%; the lowest value being recorded after their treatment was with the GHC 3-B protein gel variant at 10%. As shown in Table 6, the GHC 3-B protein gel variant recorded the lowest values of GP% (90.00–16.66%) in all three tested concentrations compared to the other two protein gel variants studied. However, a situation worth mentioning is that if we were to refer to the control, where the germination percentage is 93.33%, a higher germination percentage was recorded in the case of the GHC 1-B (100%) and GHC 2-B (96.66%) protein gel variants at a 1% concentration and at a 3% concentration of (96.66%) in the case of the GHC 1-B gel variant. This could lead us to think that these extract variants have a more balanced concentration of nutrients. In the case of tomato seeds, the GP % values fell between 100–63.33%; the lowest concentration recorded was in the case of the GHC 3-B protein gel variant at 10% concentration, and a similar situation was also found in pepper seeds, but at a much lower percentage. Moreover, in the case of tomato seeds, the percentage of germination in the control sample is lower (73.33%) compared to the protein-based gel samples taken in the study, except for the GHC 3-B variant, which was at 10% concentration. Research carried out by Casadesús et al. [30] highlights the beneficial effects of an extract based on proteins of animal origin obtained by enzymatic hydrolysis on tomato seedlings that were grown under water, and this is due, according to the study, to the influence on the increase in biosynthesis of the specific phytohormones.

#### 2.2.2. Relative Germination Index (RSG%)

The relative germination index (RSG%) is calculated by reporting the number of germinated seeds from the extract samples taken in the study to the number of germinated seeds from the control sample. Regarding the determination of RSG% values for pepper seeds, they are located between 107.14–17.85%, and in the case of tomato seeds, they fell between 122.72–86.63% (Table 7). The RSG% values in the case of tomato seeds were over 110% for all variants of hydrolyzed protein-based gels, except for the GHC 3-B variant, which was at a 10% concentration, where the RSG percentage was below 100% (86.36%). Pepper seeds recorded values over 100% only in the case of GHC 1-B protein gel variants at 1% (107.14) and 3% concentrations (103.57%) and GHC 2-B at a 1% concentration (103.57%); the rest of the values were below 100%. The researcher Milon and his collaborators [31] reported RSG % values of over 100% in the case of Napa cabbage, carrot, cabbage, and radish seeds, and in the case of tomato seeds, the RSG % values fell between 90–100% when they applied three types of compost obtained from household waste from pigs, waste obtained from charcoal processing, and compost obtained from sawdust. From our study, we can see that the level of RSG % values started to decrease both in the case of tomato seeds and in the case of pepper seeds with the increase in the concentration of hydrolyzed protein gels. We could interpret this as the fact that at higher extract concentrations, the protein extracts contain a higher amount of possible inhibitory/damaging substances, such as residual solvents left after hydrolysis processes or the presence of non-proteogenic amino acids that act as inhibitors, delaying steps or processes of the biosynthesis of amino acids or creating false signals of protein synthesis, such as in the case of ornithine [32], which was also found in all three tested protein-based gel variants. An essential thing to mention is that pepper seeds show a higher sensitivity to these compounds.

#### 2.2.3. Relative Root Growth Index

**Figure 2:**
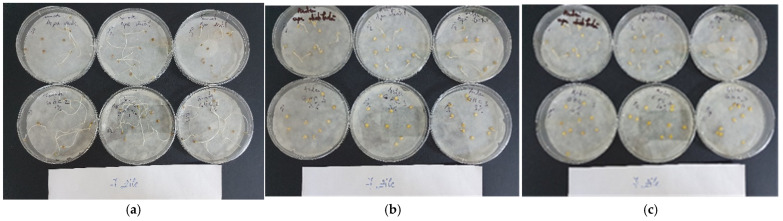
Seedling growth germination bioassay of tomato and pepper using (**a**) 1% GHC 2-B protein gel on tomato seeds in comparison with the control sample; (**b**) GHC 3-B protein gel 3% on pepper seeds in comparison with the control sample, and (**c**) GHC 3-B protein gel 10% on pepper seeds in comparison with the control sample.

The relative root growth index (RRG%), compared to the average length of germinated roots in the control sample, was between 85.24–2.68% for pepper seeds and 149.46–52.19% in the case of tomato seeds. The highest RRG value (149.46%) was statistically insignificant compared to the control sample (*p* > 0.05), which was recorded in the case of the GHC 2-B protein gel variant at a 1% concentration on tomato seeds (Table 8). The pepper seeds proved to be more sensitive after their treatment with the GHC 3-B gel variant at 3% and 10% concentrations, which recorded the lowest RRG values (19.52% and 2.62%, respectively) but also in the case of the GHC 2-B gel variant at a concentration of 3% (47.19%), which was statistically significant compared to the control sample (*p* < 0.05) (Figure 2). According to researchers Cho et al. [33], the influence of the biological substances present in the seed treatment medium is particularly evident in the development and growth of the roots because they are absorbed by the roots and have positive or negative effects on them depending of the nature and concentration of the compounds present. However, there is a relationship between the RRG% and RSG% values in tomato seeds at a 1% concentration in the case of the GHC 2-B protein gel variant because it recorded the highest values compared to the other protein gel variants tested. As is known from specialized literature [34], keratin obtained from sheep’s wool is rich in compounds that can have phytostimulating effects on plants, such as amino acids (glycine, proline, serine and cysteine, lysine, methionine, and histidine), but also sulfur and nitrogen compounds. These compounds could also be observed in the case of hydrolyzed keratin from the present study. Following the study carried out by Noroolzlo et al. [35], regarding the effect of amino acids glutamine and glycine on Romain lettuce seeds at different concentrations (250 mg/L^−1^, 500 mg/L^−1^, and 1000 mg/L^−1^), highlighted the fact that the best results regarding root development were obtained after the application of the lowest concentration of the amino acids tested. As it appears from the multitude of studies carried out previously, both glutamic acid and glutamine intervene in the process of stimulating seed germination in the growth and development of the roots of germinated seeds and, last but not least, in the process of chlorophyll synthesis. In turn, glycine intervenes in the process of tissue growth and development in the process of the synthesis of vitamins and chlorophyll. We can say that the values obtained both in the case of determining RSG and in the case of determining RRG are in close correlation with the concentration of the protein hydrolysate tested, a fact highlighted in both types of seeds tested.

#### 2.2.4. Germination Index

The determination of the germination index (Gi) is taken into account with the results obtained after the determination of RSG and RRG [36]. Following the results obtained by Gi, we can interpret whether the protein-based gels used in the study have a phytostimulant or phytotoxic effect on pepper and tomato seeds. According to Table 9, the highest Gi value (190.23%) was recorded in the case of the GHC 2-B gel variant at a 1% concentration on tomato seeds. All three variants of protein gels proved to have a phytostimulating effect at a 1% concentration, but only on tomato seeds. Gi% values were recorded as being over 100%. We cannot say this in the case of pepper seeds, where the Gi (%) values recorded for all extract variants, regardless of the concentration used, were below 100%. We can even say that at certain concentrations, the protein gels have a strong phytotoxic effect on pepper seeds, registering values of a Gi below 50%. This is evident in the case of the protein gel variant GHC 1-B at a 10% concentration (43.59%), GHC 2-B at a 3% concentration (38.68%), and GHC 3-B at 3% (13.24%) and 10% concentrations (0.47%). Such a case can also be found in tomato seeds when they were treated with the GHC 3-B gel variant at a 10% concentration, recording a Gi value of 46.91%. According to the study carried out by the researcher Perez Aguilar and his collaborators [37], on two types of hydrolyzed protein extracts of animal origin obtained by enzymatic hydrolysis of a pig carcass and household water obtained after the process of drying and the preparation of protein products (MBM), they obtained a better germination percentage for the seeds of lettuce and Chinese cabbage with the extract obtained from pork carcass, where it presented a richer concentration in the content of total amino acids (70.67%) compared to the protein extract obtained from household water produced at MBM (60.72%). As we mentioned before, the rich content of amino acids contributes, to a large extent, to the development and growth of plants. Some of them intervene in the plant development process, such as alanine, proline, arginine, and glutamic acid, some intervene and improve the nutritional intake of plants (e.g., proline, serine, and glutamic acid), others intervene in the process of the production of chlorophyll by improving it (alanine, arginine, glycine and glutamic acid), and another part intervenes in the process of reducing the stress produced by the presence of salinity in the environment [38].

Another study carried out by Berechet and his collaborators [39] highlighted the fact that the mixture of keratin obtained by alkaline hydrolysis and the application of heat treatment (85 °C), which had a high nitrogen content in the composition (14.39%), protein content of 87.10%, and low particle size, was tested as a possible fertilizer at 3% and 5% concentrations on two types of wheat seeds; it had a better fertilizing effect at the 5% concentration. The length of the roots of the germinated seeds was recorded as being significantly higher for both types of seeds compared to the control sample.

## 3. Conclusions

Four types of protein hydrolysates were obtained by acid–thermal, alkaline, and alkaline–enzymatic hydrolysis. These, in turn, were combined in different proportions, finally obtaining three variants of protein gel. Following the hydrolysis methods, the goal was to obtain a balanced content of the main bioactive compounds (amino acids and peptides) with stimulating properties on the plants. At the same time, the current study reveals information regarding obtaining future products with stimulating properties that are based on protein hydrolysates that are obtained from waste left after the processing of the leather industry, bringing a positive contribution to the purpose of the development of ecological manufacturing technologies. According to the obtained results, it was demonstrated that all three protein gel variants showed a stimulant effect only on tomato seeds at the minimum concentration applied because these types of mixtures are present in their composition substances with a phytonutrient effect only for certain categories of plants. It has been demonstrated [40] that too high a concentration of amino acids (phenylalanine, valine, and proline) could inhibit plants. This could also explain the results we obtained. Protein gels presented a phytostimulating effect only at a 1% concentration. On the other hand, pepper seeds proved to be more sensitive to substances of biological origin (from the composition of the hydrolyzed product) and chemicals (residual materials after hydrolysis—salts, acids, and bases) from the composition of the protein gels. This was observed following the delayed process of seed germination, inhibiting the growth and development of the roots at the same time. As demonstrated in the specialized literature, but also according to the results obtained from our study, biostimulators based on protein hydrolysates, depending on their biochemical composition, can be used both for foliar application, by treating the soil of the plants, or even for seed treatment [41].

## 4. Materials and Methods

### 4.1. Materials Used in Obtaining and Characterizing Protein Hydrolysates

The following chemical materials were used in this study: residual semiprocessed bovine leather was collected from the leather processing pilot station of INCDTP—Division Leather and Footwear Research Institute—chopped, and preserved by freezing. Wool was purchased from sheep farmers and degreased at the INCDTP Division Leather and Footwear Research Institute. Hydrated calcium oxide (CaO CaOH, MW = 81.371 g/mol) was purchased from Cristal R Chim SRL (Bucharest, Romania). Analytical grade chemical reagents, such as ammonia potassium hydroxide and oxalic acid, were purchased from Chimreactiv SRL (Bucharest, Romania). Alcalase 2.4 L (protease from Bacillus licheniformis with 2.4 U/g activity) and propionic acid were purchased from Sigma-Aldrich (Bucharest, Romania). Protamex^®^ (an endo-protease from *Bacillus* spp. with 1.5 U/g activity) was purchased from Novozymes (Atasehir, Turkey).

### 4.2. Extraction Methods and Characterization of Protein Hydrolysates

#### 4.2.1. Gelatin and Collagen Hydrolysate Preparation from Residual Untanned Leather Fragments

Gelatin (GPU-B) and collagen hydrolysates (HPU-B) from residual untanned leather fragments were prepared by thermal and enzymatic hydrolysis. Residual semiprocessed bovine leather fragments were mixed with a certain amount of water, and the pH of the dispersion was adjusted between 5.5 and 6 using a solution of 1 M propionic acid. The dispersion was heated at 80 °C for six hours, and the pH was controlled and adjusted hourly. The gelatin solution was then filtered and dried in an exhaust oven at 60 °C. The residues from gelatin extraction were hydrolyzed in water with Alcalase 2.4 L for 2 h at a temperature of 60 °C under mechanical stirring. After enzyme deactivation at 90 °C, the mass reaction was cooled static for 16–18 h, the pH was adjusted to 8.0–9.0 value with 25% tartaric acid solution, and the reaction mass was heated at 60 °C and 1.0% Alcalase 2.4 L (*w*/*w*) was added under stirring for 2 h at 60 °C. After 2 h of enzymatic hydrolysis, the temperature was raised to 90 °C and maintained for 15 min until enzyme deactivation. After filtration, the liquid collagen hydrolysate was collected and dried by forced convection at a temperature of 60 °C, after which the dry matter was ground.

#### 4.2.2. Collagen Hydrolysate Preparation from Residual Tanned Leather Fragments

The collagen hydrolysate, noted with HCE-B, was prepared by alkaline and enzymatic hydrolysis of residual bovine-tanned leather fragments. The residual bovine-tanned leather fragments were hydrated at a 1:5 (*w*/*v*) ratio and mixed with hydrated calcium oxide in a stainless-steel vessel equipped with a mechanical stirrer and automatic temperature control at 80 °C, for 6 h. After vacuum filtration and pH = 8.5–9.0 adjustment with 1N propionic acid solution the mass reaction was enzymatically hydrolyzed with Alcalase 2.4 L for 2 h at 60 °C. For enzyme deactivation, the temperature was increased to 90 °C and maintained at this temperature for 15 min. After decanting, the liquid collagen hydrolysate was dried by forced convection at 60 °C. After cooling, the dry matter was ground.

#### 4.2.3. Keratin Hydrolysate Preparation from Degreased Residual Wool

Keratin hydrolysate was noted with HKU-B and prepared by alkaline and enzymatic hydrolysis from degreased residual ovine wool. The degreased residual ovine wool was chopped, hydrated 1:20 (*w*/*v*), bleached with hydrogen peroxide (30% solution), and mixed with potassium hydroxide and hydrated calcium oxide in a stainless-steel vessel equipped with a mechanical stirrer and automatic temperature control at 80 °C, for 4 h, after remaining static for 16–18 h. After pH = 8.0, with 30% oxalic acid solution, the mass reaction was enzymatically hydrolyzed with Protamex for 2 h at 60 °C. For enzyme deactivation, the temperature was increased to 90 °C and maintained at this temperature for 15 min. After vacuum filtration, the liquid keratin hydrolysate was dried by forced convection at 60 °C, after which the dry matter was ground.

#### 4.2.4. Preparation of Protein Combination

To prepare a product with nutritional properties for plants, three protein-based gels were prepared from gelatin, hydrolysates of collagen, and keratin extracted according to the processes described in Section 4, in the proportions described in Table 10. Homogenization of protein-based gels was achieved by stirring for one hour at 50 °C.

#### 4.2.5. Gelatin, Collagen, and Keratin Hydrolysates Characterization

The physical–chemical characteristics were analyzed according to standardized methods for volatile matter [42], ash content [43], total nitrogen and protein content [44], pH [45], cysteine and cystine sulfur [46], and aminic nitrogen [47]. The results of the analyses are expressed as the average values of three determinations. Texture properties of gelatin (strength, consistency, elasticity, adhesion force) were analyzed according to GMIA standard methods [48] for the testing of edible gelatin (Official Procedure of the Gelatin Manufacturers Institute of America, Inc., Muscatine, IA, USA), using a TEX’AN TOUCH Texture Analyser, equipped with Bloom cylinder and special cylinder for CRT (Compression-Relaxation-Traction). Structural analysis of collagen extracts as gelatin and hydrolysate was performed using a double-beam IR molecular absorption spectrometer in the range 4000–400 cm^−1^, using the FT-IR Thermo Nicolet iS 50 (Thermo ScientificTM, Waltham, MA, USA). The amino acid composition of gelatin, collagen, and keratin hydrolysates was analyzed by HPLC using an Amino Acid Analyzer LC 3000 (Sykam GmbH, Eresig, Germany), equipped with a polymeric cation exchanger column, post-column ninhydrin derivatization at 125 °C, and photometric measurement at 570 nm. The results were monitored by Chromatography-Software ChromStar 6.0 (SCPA GmbH, Bremen, Germany) and were reported as a means of triplicate determinations. The surface properties of the protein combinations were determined at a temperature of 28 °C with a Sigma 703D Force Tensiometer (Dyne Testing Ltd., Staffordshire, UK) for the surface tensions and with a DataPhysics Instrument OCA 25 (DataPhysics Instruments, Stuttgart, Germany) for contact angle measurement in static mode. The nanometric particle size and distribution in the protein combination were analyzed by Dynamic Light Scattering (DLS) using a ZetaSizer Nano ZS (Malvern, UK). The determinations were made using solutions containing 1% protein.

#### 4.2.6. Seed Germination Bioassay

To determine the phytostimulant potential of the protein-based gels GHC 1-B, GHC 2-B, and GHC 3-B by applying the germination bioassay, commercial seeds of tomato, variety ACE55VF, and pepper seeds, variety Alexandru were used. The method used was modified and adapted according to the procedures reported by researchers Miteluț and Popa [17], Ghayal et al. [49], and Perisoara et al. [50]. All three protein gel variants were studied at concentrations of 1%, 3%, and 10%, diluted with distilled water, and the results obtained were compared to the negative control, which contains distilled water. Petri plates (Corning Petri plates), with a diameter of 100 mm × 200 mm, were disinfected with isopropyl alcohol 70% (Contec IPA filtered 70% isopropanol) and left to dry, filter paper (IDL GMBH, type blue) with a diameter of 90 mm, was sterilized in a hood, with a UV lamp, for 60 min, pepper and tomato seeds were washed with purified water and dried in an oven at 30 °C. After completing the sterilization process of the filter papers, they were placed in Petri dishes, on which 10 seeds of ACE55VF tomato variety/Alexandru pepper variety of close size were placed separately and were pipetted 5 mL of the samples obtained after dilutions. The whole experiment was performed in three batches, each with 10 seeds. The Petri plates were left to incubate in the dark for 7 days at a temperature of 25 °C ± 1. At the end of the incubation period, germinated seeds were counted, denoted by G, and the root length, denoted by L, was measured. The results obtained were analyzed by determining the following indicators: the percentage of germination, denoted by GP, the index relative germination of the seeds—RSG—the relative growth index of the roots—RRG—and the germination index—Gi—using the following calculation formulas [34,51]:

(1) $$\mathrm{GP}=\frac{Number\;of\;germinated\;seeds}{Total\;number\;of\;seeds}\;\times \;100\%,$$

(2) $$\mathrm{RSG}=\frac{Number\;of\;germinated\;seeds\left(sample\right)}{Total\;number\;of\;seeds\left(control\right)}\;\times \;100\%;$$

(3) $$\mathrm{RRG}=\frac{Root\;length\;of\;germinated\;seeds\left(sample\right)}{Root\;length\;of\;germinated\;seeds\left(control\right)}\;\times \;100\%;$$

(4) Gi=(GGc) × (LLc) × 100%

*G*—represents the number of germinated seeds on the sample substrate,
*Gc*—represents the number of germinated seeds in the control,
*L*—is the average length of plant roots per substrate sample,
*Lc*—is the average length of plant roots on the control substrate.

According to specialized literature [52,53], the registration of a value of Gi < 50% indicates high phytotoxicity of the substance under study, the setting of the value of Gi between 50–80% indicates moderate toxicity, Gi ˃ 80%, the substances under study are free of phytotoxic, recording a Gi value close to 0 indicates extreme phytotoxicity, and when the germination index exceeds 100%, the extract is considered to be a very good phytonutrient or phytostimulant for the tested seeds. The incubation period will end when at least 65% of the seeds in the control sample have germinated and developed roots at least 20 mm long [54,55].

## 5. Statistical Analysis

The germination study was carried out in triplicate; the results were reported as a mean and standard deviation (±), and the relative standard deviation (RSD) was calculated. The statistical analysis was carried out using Microsoft Excel 2019. The results were interpreted by applying the well-known Single Factor Anova to compare the activity of gels based on protein hydrolysates regarding the activity from the germination study. A value of *p* < 0.05 was considered to be statistically significant.

## Figures and Tables

**Table 1:** The physicochemical characteristics of the protein extracts and their combination.

Characteristics	GPU-B	HPU-B	HCE-B	HKU-B	GHC 1-B	GHC 2-B	GHC 3-B
Volatile matter (%)	6.49	7.27	6.19	7.54	12.58	12.65	12.53
Total ash (%)	2.50	7.39	5.98	7.80	6.04	7.83	7.50
Total nitrogen (%)	14.84	13.68	14.01	12.60	16.69	14.78	15.16
Protein content (%)	83.42	76.88	78.74	76.35	93.82	83.08	85.22
Aminic nitrogen (%)	0.50	0.60	0.99	0.62	0.76	0.48	0.75
Cysteine, %	-	-	-	8.20	-	-	-
Cystinic sulphur, %	-	-	-	2.19	-	-	-
pH	5.08	8.04	8.24	7.68	8.02	7.80	7.71

**Table 2:** The amino acid content of the protein extracts.

Amino Acid (%)	GPU-B	HPU-B	HCE-B	HKU-B
Cys(O3H)	0.00	0.00	0.00	1.11
Asp (D/N)	5.52	5.33	5.31	7.40
Hyp	8.04	9.48	8.96	0.00
Thr (T)	1.79	1.10	0.79	4.30
Ser (S)	2.31	1.72	1.09	4.54
Glu (E/Q)	10.70	10.08	10.44	16.95
Pro (P)	15.23	14.84	15.06	7.55
Gly (G)	20.31	24.51	24.85	7.11
Ala (A)	9.92	10.27	9.90	6.01
Val (V)	2.43	2.52	2.54	8.13
Cys	0.24	0.00	0.16	0.84
Met (M)	0.92	0.92	1.01	0.77
Ile (+ allo-Ile) (I)	1.60	1.54	1.49	3.97
Leu (L)	3.12	2.79	2.84	9.34
Tyr (Y)	0.48	0.43	0.25	3.63
Phe (F)	2.07	1.92	1.96	4.00
deg. Prod. Trp (W)	0.72	0.00	0.00	0.00
His (H)	0.66	0.77	0.72	0.85
Hyl	0.77	0.84	0.85	1.06
Ornithine	0.00	0.59	1.27	0.00
Lys (K)	3.64	3.00	3.12	2.51
NH3	0.51	0.52	0.60	0.89
Arg (R)	9.02	6.85	6.80	9.05
Total	100.00	100.0	100.00	100.00

**Table 3:** The surface tensions of protein-based gel combinations.

Sample	Surface Tension (mN/m)				
	Test I	Test II	Test III	Test I|V	Average
Control	68.45	68.39	68.21	68.35	68.45
GHC 1-B	57.85	57.32	58.00	57.72	57.85
GHC 2-B	52.73	53.24	52.24	52.74	52.73
GHC 3-B	59.90	59.66	57.79	59.12	59.90

**Table 4:** The contact angles of protein-based gel combinations.

Sample	Contact Angles (Degree)				
	Test I	Test II	Test III	Test I|V	Average
Control	49.9	45.8	49.5	-	48.40
GHC 1-B	38.3	44.8	52.0	42.3	44.35
GHC 2-B	43.9	40.8	48.3	-	44.33
GHC 3-B	43.9	43.6	45.8	-	44.43

**Table 5:** The size distribution of nanometric particle populations.

Sample	Weight of Particle Size (%)			
	0–10 nm	10–100 nm	100–1000 nm	1000–6000 nm
GHC 1-B	5.2	12.5	47.0	35.3
GHC 2-B	-	-	6.9	93.1
GHC 3-B	-	-	60.9	39.1

**Table 6:** Germination percentage GP% on tomato and pepper seeds after treatment with hydrolyzed protein-based gels.

**Name of the Sample Applied as a Treatment on Pepper Seeds**	**% Sample/Control**			
	**1%**	**3%**	**10%**	**Control**
GHC 1-B	100.00 ± 0.00 RSD = 0.00	96.66 ± 0.57 RSD = 5.97	70.00 ± 1.73 RSD = 24.74	93.33 ± 1.15 RSD = 12.37
GHC 2-B	96.66 ± 0.57 RSD = 5.97	76.66% ± 1.52 RSD = 19.92	80.00 ± 1 RSD = 12.50	
GHC 3-B	90.00 ± 1 RSD = 11.11	63.33 ± 1.15 RSD = 18.23	16.66 ± 1.15 RSD = 69.28	
**Name of the sample applied as a treatment on tomato seeds**	**% sample/control**			
	**1%**	**3%**	**10%**	**Control**
GHC 1-B	90 ± 1 RSD = 11.11	83.33 ± 0.57 RSD = 6.92	83.33 ± 0.57 RSD = 6.92	73.33 ± 0.57 RSD = 7.87
GHC 2-B	93.33 ± 0.57 RSD = 6.18	86.66 ± 2.30 RSD = 26.64	86.66 ± 0.50 RSD = 5.87	
GHC 3-B	86.66 ± 1.15 RSD = 13.32	83.33 ± 2.08 RSD = 24.97	63.33 ± 0.57 RSD = 9.11	

**Table 7:** RSG% relative germination index on tomato and pepper seeds after treatment with hydrolyzed protein gels.

**Name of the Sample Applied as a Treatment on Pepper Seeds**	**% Sample/Control**			
	**1%**	**3%**	**10%**	**Control**
GHC 1-B	107.14	103.57	75.00	-
GHC 2-B	103.57	82.14	85.71	
GHC 3-B	96.42	* 67.85	* 17.85	
**Name of the sample applied as a treatment on tomato seeds**	**% sample/control**			
	**1%**	**3%**	**10%**	**Control**
GHC 1-B	122.72	122.72	113.63	-
GHC 2-B	* 127.72	118.18	* 118.18	
GHC 3-B	118.18	113.63	86.36	

* *p* < 0.05 statistically significant.

**Table 8:** Relative root growth index—RRG %—on tomato and pepper seeds following treatment with hydrolyzed protein gels.

**Name the Sample Applied as a Treatment on Pepper Seeds**	**% Sample/Control**			
	**1%**	**3%**	**10%**	**Control**
GHC 1-B	57.43% (** 6.42 mm ± 1.28 RSD = 19.98)	71.53% (8.00 mm ± 0.87 RSD = 10.98)	58.12% (6.5 mm ± 1.67 RSD = 25.69)	11.18 mm ± 3.39 RSD = 30.35
GHC 2-B	85.24% (9.53 mm ± 0.77 RSD = 8.147)	* 47.09% (5.26 mm ± 1.30 RSD = 24.70)	63.18% (7.06 mm ± 1.65 RSD = 23.35)	
GHC 3-B	60.20% (6.73 mm ± 1.38 RSD = 20.59)	* 19.52% (2.18 mm ± 0.45 RSD = 20.77)	* 2.68% (0.30 mm ± 0.34 RSD = 115.47)	
**Name of the sample applied as a treatment on tomato seeds**	**% Sample/Control**			
	**1%**	**3%**	**10%**	**Control**
GHC 1-B	98.40% (43.3 mm ± 14.68 RSD = 33.92)	64.84% (28.53 mm ± 13.80 RSD = 48.36)	63.25% (27.83 mm ± 2.23 RSD = 8.03)	44.00 mm ± 15.23 RSD = 34.63
GHC 2-B	149.46% (65.76 mm ± 2.21 RSD = 3.37)	77.27% (34.00 mm ± 20.51 RSD = 60.33)	52.19% (22.96 mm ± 1.79 RSD = 7 81)	
GHC 3-B	86.51% (38.06 mm ± 8.67 RSD = 22.79)	78.25% (34.43 mm ± 17.32 50.31)	54.31% (23.90 mm ± 4.55 RSD = 19.06)	

** Average root length of germinated seeds expressed in mm. * *p* < 0.05 statistically significant compared to the control sample.

**Table 9:** Germination index—Gi % on tomato and pepper seeds after treatment with hydrolyzed collagen protein extracts.

**Name the Sample Applied as a Treatment on Pepper Seeds**	**% Sample**		
	**1%**	**3%**	**10%**
GHC 1-B	61.53	74.08	43.59
GHC 2-B	88.29	38.68	54.16
GHC 3-B	58.05	13.24	0.47
**Name of the sample applied as a treatment on tomato seeds**	**% sample**		
	**1%**	**3%**	**10%**
GHC 1-B	120.77	73.69	71.88
GHC 2-B	190.23	91.32	61.68
GHC 3-B	102.24	88.92	46.91

**Table 10:** The composition of protein combinations.

Compositions	Gelatin (%) GPU-B	Collagen Hydrolysates (%)		Keratin Hydrolysate (%) HKU-B
		HPU-B	HCE-B	
GHC 1-B	20	-	80	-
GHC 2-B	20	-	40	40
GHC 3-B	20	80	-	-

## Data Availability

The data presented in this study are openly available in article.
